# Impact of the robotic-assistance level on upper extremity function in stroke patients receiving adjunct robotic rehabilitation: sub-analysis of a randomized clinical trial

**DOI:** 10.1186/s12984-022-00986-9

**Published:** 2022-02-25

**Authors:** Takashi Takebayashi, Kayoko Takahashi, Yuho Okita, Hironobu Kubo, Kenji Hachisuka, Kazuhisa Domen

**Affiliations:** 1 Department of Occupational Therapy, School of Comprehensive Rehabilitation, College of Health and Human Sciences, Osaka Prefecture University, 3-7-30, Habikino, Osaka 583-8555 Japan; 2 Department of Occupational Therapy, School of Allied Health Science, Kitasato University, Kanagawa, Japan; 3 Faculty of Health, Arts and Design, Swinburne University of Technology, Melbourne, Australia; 4 Department of Medical Science, Teijin Parma Limited, Tokyo, Japan; 5 Moji Medical Center, Fukuoka, Japan; 6 Department of Rehabilitation Medicine, Hyogo College of Medicine, Hyogo, Japan

**Keywords:** Occupational therapy, Robotics, Stroke

## Abstract

**Background:**

Robotic therapy has been demonstrated to be effective in treating upper extremity (UE) paresis in stroke survivors. However, it remains unclear whether the level of assistance provided by robotics in UE training could affect the improvement in UE function in stroke survivors. We aimed to exploratorily investigate the impact of robotic assistance level and modes of adjustment on functional improvement in a stroke-affected UE.

**Methods:**

We analyzed the data of 30 subacute stroke survivors with mild-to-severe UE hemiplegia who were randomly assigned to the robotic therapy (using ReoGo System) group in our previous randomized clinical trial. A cluster analysis based on the training results (the percentage of each stroke patient’s five assistance modes of robotics used during the training) was performed. The patients were divided into two groups: high and low robotic assistance groups. Additionally, the two groups were sub-categorized into the following classes based on the severity of UE functional impairment: moderate-to-mild [Fugl-Meyer Assessment (FMA) score ≥ 30] and severe-to-moderate class (FMA < 30). The outcomes were assessed using FMA, FMA-proximal, performance-time in the Wolf motor function test (WMFT), and functional assessment scale (FAS) in WMFT. The outcomes of each class in the two groups were analyzed. A two-way analysis of variance (ANOVA) was conducted with robot assistance level and severity of UE function as explanatory factors and the change in each outcome pre- and post-intervention as the objective factor.

**Results:**

Overall, significant differences of the group × severity interaction were found in most of the outcomes, including FMA-proximal (p = 0.038, η^2^ = 0.13), WMFT-PT (p = 0.021, η^2^ = 0.17), and WMFT-FAS (p = 0.045, η^2^ = 0.14). However, only the FMA score appeared not to be significantly different in each group (p = 0.103, η^2^ = 0.09).

**Conclusion:**

An optimal amount of robotic assistance is a key to maximize improvement in post-stroke UE paralysis. Furthermore, severity of UE paralysis is an important consideration when deciding the amount of assistance in robotic therapy.

*Trial registration* Trial enrollment was done at UMIN (UMIN 000001619, registration date was January 1, 2009)

## Background

In terms of the general background of the stroke rehabilitation, hemiplegia of the upper extremity (UE) is one of the most encountered conditions in hospital-admitted stroke survivors, affecting two-thirds of this population. Hemiplegia of the UE causes functional impairment of the arm and hand, thus negatively impacting the daily activities of stroke survivors [1, 2]. Rehabilitation therapy is considered the foundation of stroke treatment for improving the motor skills and quality of life of survivors. Further, repetitive training is an effective method to facilitate recovery from stroke and assist in restructuring the neural networks [3, 4].

Therefore, in stroke rehabilitation, robots have been widely utilized by clinicians, as they allow the user to perform repetitive movements consistently and precisely [5]. Robotic therapy enables patients to perform repetitive training with voluntary movements by providing mechanical assistance to the upper limbs, which can hardly move voluntarily due to the sequelae of stroke. In stroke rehabilitation, “active assistive training” is a traditional method that has been employed in many clinical settings (the patient attempts a voluntary movement while the therapist provides some form of limb support and mechanical assistance to complete the desired movement).

Reportedly, in terms of robotic therapy in patients with stroke, the guidelines stipulated by the American Heart/Stroke Association are effective for the treatment of UE paresis in stroke survivors [6]. However, whether robotic therapy can produce better UE recovery compared to conventional rehabilitation remains unclear. Further, a previous randomized controlled trial (RCT) showed no significant difference between conventional rehabilitation and robotic therapy in UE functionality improvement in stroke survivors [7–9]. A systematic review also indicated that each robotic therapy and a high-intensity conventional approach produced similar effects on UE improvement in stroke survivors [10]. Additionally, another systematic review and meta-analysis concluded that robotic therapy could produce more significant improvement than active assistive training, but the effect size of robotic therapy appeared to be relatively small [11].

In the study of robotic therapy applications, one of the most developed paradigms is the investigation of robot-generated assistance. Recent robotic therapy using assistive controllers, such as force-field, intelligence difficulty (the exercise via audiovisual) [12], and feedforward controllers, allow stroke survivors to move their affected UE more independently. This exercise could be conducted in a similar way as “active assist” exercise, which is manually assisted by a rehabilitation therapist. The developed rehabilitation robot behaviour mode is composed of two concepts: error reduction and error augmentation. The error reduction concept indicates that the use of robot assistance can reduce the risk of movement error occurrence by allowing the performance of appropriate exercises, which can reinforce the process of motor learning [13, 14]. In contrast, the error augmentation concept stipulates that, to exaggerate movement errors, challenging-based assistive controller should be used in resistance training, which facilitates the process of motor learning [15]. Considering the nature of robotic treatment according to the two different types of concepts mentioned above, robotic treatment program in stroke rehabilitation has become more complex in recent years.

Some previous studies recommend the error reduction strategy using robotic assistance in UE training for improving UE function in stroke survivors. Rowe et al. [16] investigated the appropriate amount of robotic assistance using finger exoskeleton robotics and found that greater robotic assistance improved the UE function on the affected side compared to less robotic assistance in post-stroke practice (there was no significant difference, but there was a significant improvement in the secondary outcomes). On the other hand, several researchers have reported the possibility of negative effects from physical active assistive exercises [17, 18]. These studies suggest that excessive robotic assistance in intentional movements may contribute to the Slacking Hypothesis (decreased motor output, energy expenditure, and attention performed by the subject during the exercise). This would highlight the importance of providing “assistance-as-needed” in active assistive exercises [15]. These findings suggest that it remains unclear whether a positive rehabilitation outcome correlates with the level of robotic assistance provided for a stroke survivor to complete UE voluntary movement training.

Therefore, the purpose of this exploratory study was to examine the trend of the impact of robotic assistance level in order to derive data for future prospective controlled trial. In this paper, we defined “the level of robotic assistance” as “the amount of physical support generated by a robot when performing an active assistive exercise”. “Robotic therapy” was defined as a therapy using robotic assistance to improve the affected UE function of stroke patients with different severities of UE paralysis.

## Methods

### Study design and approval

This study utilized secondary analysis data from our previously published multicenter, randomized, open-label, blinded-endpoint, clinical trial and followed its protocol [19], which was approved by the institutional review boards of each of the six participating institutions. This study was conducted in accordance with the tenets of the Declaration of Helsinki, and all participants in this study provided written informed consent before their inclusion in the research. Details of the study protocol can be found as supplemental data of the previous study [19].

### Participants

Here, we recruited patients from the inpatient stroke centers of six rehabilitation hospitals in Japan from November 2008 to April 2010. The following inclusion criteria were used to select the participants in this study: age range 20–80 years; first stroke (ischemic or hemorrhagic stroke) patients diagnosed by a neurosurgery/neurology doctor unrelated to this study; an interval of 4–8 weeks from the day of stroke onset to its confirmation using magnetic resonance imaging or computed tomography; currently experiencing UE hemiplegia with Brunnstrom recovery stages III or IV; and hospitalization in the rehabilitation unit throughout the intervention.

The following patients were excluded: patients with (a) brainstem stroke, (b) hemorrhagic cerebral infarction or subarachnoid hemorrhage, (c) a vision disorder, (d) severe aphasia, (e) other neuromuscular disorders, f) previous experience of robot-assisted rehabilitation, functional electrical stimulation therapy, or constraint-induced movement therapy for UE hemiplegia (before participating in this study, we assumed the exclusion of patients with the first-onset stroke who received robotic therapy within 3 weeks of stroke onset), (g) overweight (weight ≥ 110 kg), (h) cardiac or pulmonary disorders that could affect rehabilitation, (i) limited capacity to remain seated during the intervention, (j) severe pain when external pressure is applied to the involved UE, and (k) limited ability to provide voluntary informed consent.

Overall, 715 patients from the assigned hospitals underwent the screening process, and 60 patients were selected based on the selection criteria. Randomization was implemented for the participants who received either robotic therapy (robotic group, N = 30) or self-guided therapy (self-guided group, N = 30), in addition to standard rehabilitation. Here, we included 30 stroke patients from the robotic group in a previous RCT to investigate the appropriate level of robotic assistance for the improvement of the affected UE function.

### Intervention

**Fig. 1:**
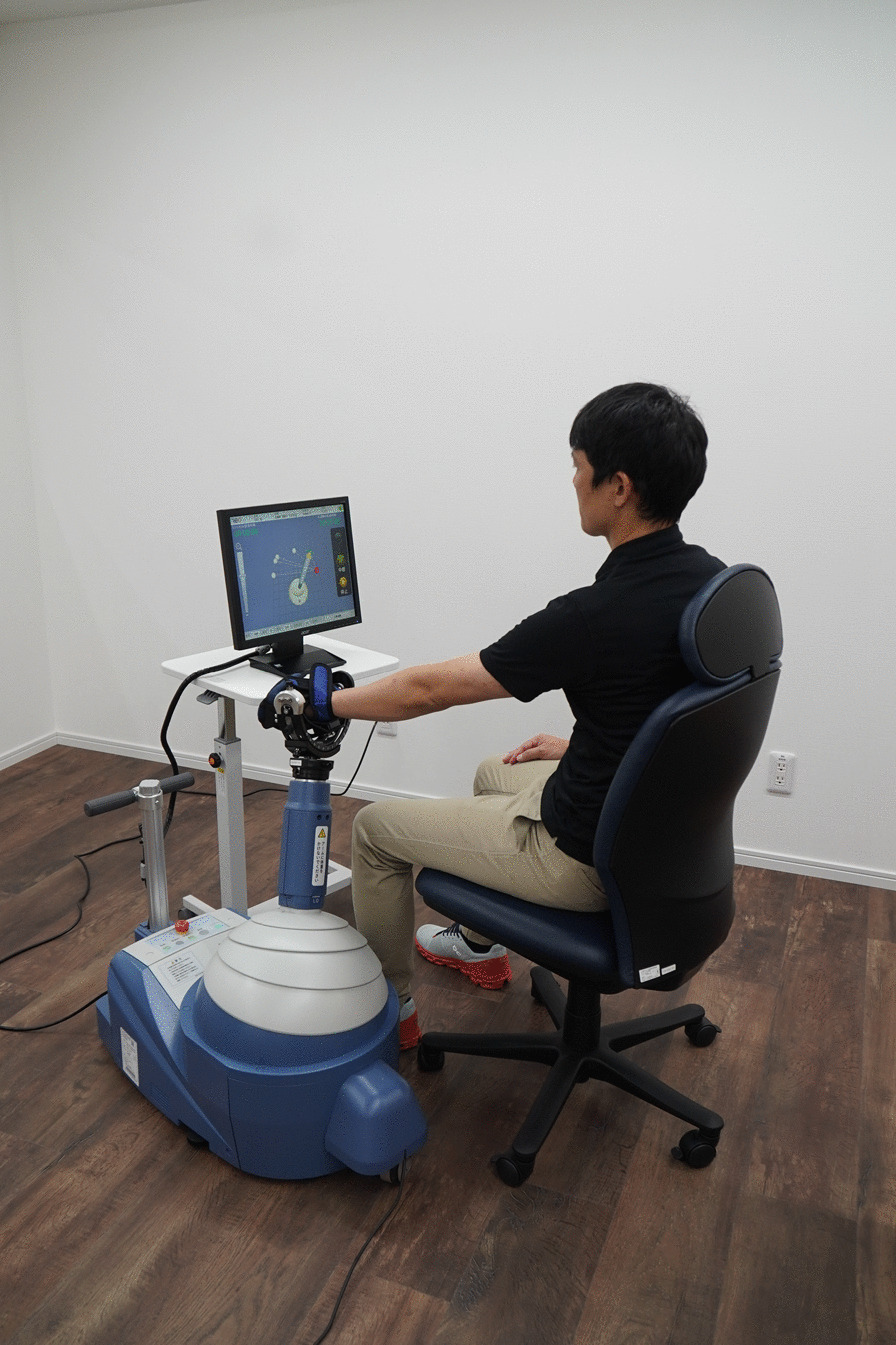
Robot-assisted self-training using the ReoGo upper extremity rehabilitation device (Teijin Pharma Ltd., Tokyo)

**Fig. 2:**
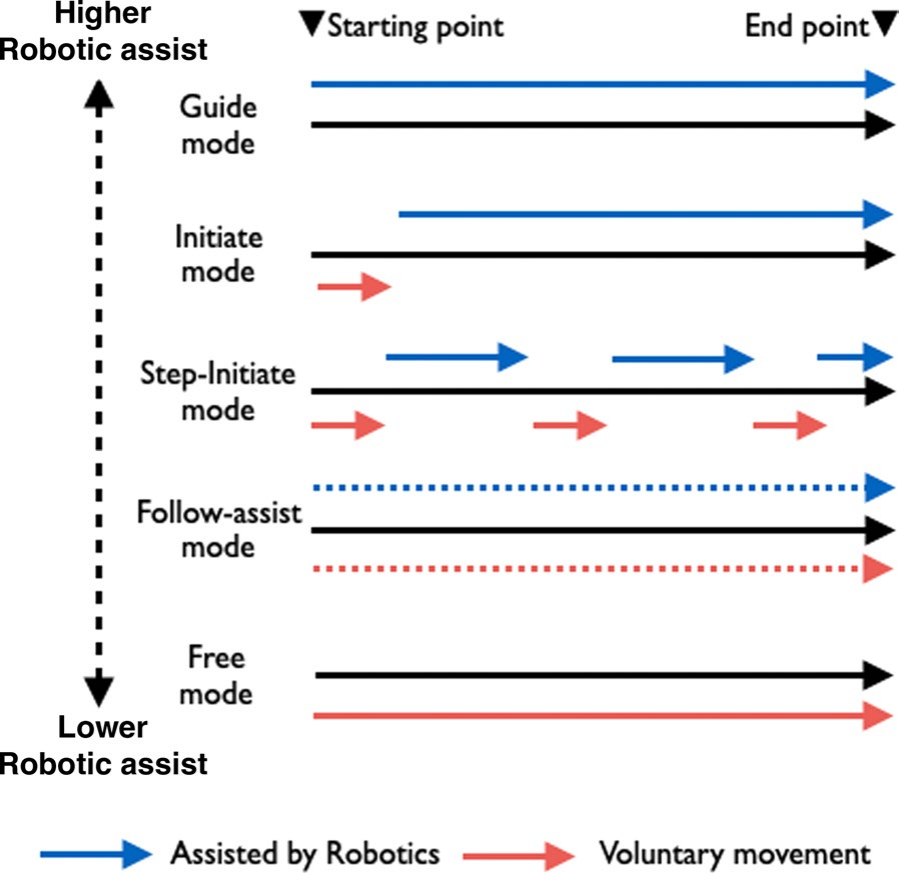
Robotic assistance at the different modes produced by ReoGo for voluntary movement. Guided mode: fully dependent on robotic assistance to complete training. Initiated mode: requires voluntary movement only at the beginning of training; for the remainder of the training, full dependence on robotic assistance is required. Step-initiated mode: requires only a few voluntary movement and robot-dependent movement alternately to complete the training. Follow-assist mode: required above a certain level of voluntary movement in training while receiving low level of robotic assistance continuously. Free mode: uses voluntary movement to complete the training without the requirement of robotic assistance

Experienced therapists with national certification as occupational therapists, who were able to modify each therapy based on the participant’s needs, provided the participants with a conventional UE rehabilitation therapy for 40 min/day over the 6-week study duration. This therapeutic duration was selected based on the commonly used, standard duration of therapy for the subacute phase after a stroke in Japan. In robotic group, participant received robotic therapy (Figs. 1, 2) for 40 min/day, in addition to conventional therapy, over the 6-week period (details of the interventions are described in our previous study) [19].

The participants were not allowed to receive constraint-induced therapy, functional electrical stimulation therapy, physical or occupational therapy of UE > 80 min/day, or any other robotic therapy for UE rehabilitation. All participants could access pharmacotherapy, patient-initiated self-training, and any type of therapy or training for the non-affected UE.

### Efficacy outcome measures

A blinded therapist, who was not involved in the participant selection process, administered the Fugl-Meyer Assessment (FMA) and Wolf Motor Function Test (WMFT) before and after the 6-week intervention. The FMA was used to measure performance-based impairment in the participants, which was due to hemiplegia caused by stroke, using a multi-item scoring system [20]. The following FMA components were used to measure the research outcome: total UE motor score (range, 0–66 points) and proximal UE score (0–36 points). WMFT was used to assess the performance of the affected UE via the following components: mean time (0–120 s) and the functional assessment scale (FAS), using a 6-point Likert scale (0–5 points), in 15 tasks [21].

### Statistical analysis

This sub-analysis specifically targeted the 30 participants who were assigned to the robotic therapy group. In this study, all study analyses were completed by an individual who was not involved in the study process, including the participant selection and intervention processes.

In this study, we conducted a cluster analysis for 30 participants assigned to the robotic therapy group. In this cluster analysis, the robot automatically acquired the log data of ReoGo’s five assistance modes (Guided mode, Initiated mode, Step-Initiated mode, Follow-assist mode, and Free mode) pre- and post-intervention in all subjects. The subjects were then classified into two groups on the basis of cluster analysis using the number of times in each mode was used as an explanatory variable.

Patients who underwent prolonged training using modes with high robotic assistance (the “Guide” and “Initiate” modes) and little robotic assistance (the “Step-initiated,” “Follow-assist,” and “Free” modes) were categorized into the high- and low robot assistance groups, respectively. This cluster analysis was conducted with agglomerative hierarchical clustering, with the following steps: (1) forming clusters about each target observation component and calculating the dissimilarities between clusters; (2) clustering the two most similar clusters based on the findings from step (1); (3) calculating the dissimilarities between the newly formed clusters in step (2) and the existing clusters, and calculating the dissimilarities among all clusters; and (4) repeating steps (2) and (3) until all observed components form one cluster. Thus, this is a method in which one observation joins individual clusters sequentially to generate a hierarchy of clusters. For the dissimilarities (distance) between the observations, we combined the two most similar observations using the Euclidean distance.

Ward’s method was utilized to calculate the dissimilarities in each cluster. This is a method of forming clusters based on the criterion of maximizing the ratio of the variance within a group to the variance between groups when two clusters are fused together. An unpaired t-test was used to identify differences in the levels of each assist mode. We sought to investigate whether there were similarities between the low and high robotic assistance groups. We also adopted Ward’s method because it is robust to outliers and tends to form clusters of equal numbers. The preparatory analysis mentioned above was described in “Clinical setting (participant flow and characteristics)” subsection of “Methods”.

The two groups formed by the cluster analysis (high and low robotic assistance groups) were sub-categorized into two classes based on their FMA scores, which are as follows: severe-to-moderate class (baseline FMA score < 30) and moderate-to-mild class (baseline FMA score ≥ 30). The Shapiro–Wilk test was used to confirm the normalization of the change in each outcome pre- and post-intervention for all groups and classes. When the normalization is confirmed, we then confirmed whether the level of robotic assistance (group) in each severity would affect the change of each outcome. A two-way analysis of variance (ANOVA) was performed to determine the mean difference of each outcome change pre- and post-intervention in subjects with different classes in the high and low robotic assistance groups. Therefore, in this analysis, we considered the amount of change in each outcome pre- and post-intervention to be the objective variable and the difference in the level of robotic assistance (group) and severity (class) as the independent variables. For the effect size of the two-way ANOVA, we calculated η^2^, and the interpretations of η^2^ is as follows: 0.01 (small); 0.06 (medium); and 0.14 (large).

However, this statistical analysis did not perform multiple comparisons (adjustment for multiplicity) of multiplicity by repeating the tests. To indicate statistically significant differences (p < 0.05), the SAS ver. 9.4 (SAS institute Inc., Cary, North Carolina) software was utilized for all statistical analyses in this study. Additionally, Cluster analysis was conducted via the clustering procedure or tree procedure of SAS (ver. 9.4).

### Clinical setting (participant flow and characteristics)

Thirty participants in the robotic group completed the 6-week intervention program. The participant flow is presented in Fig. 1, which followed the CONSORT 2010 [22] diagram.

**Fig. 3:**
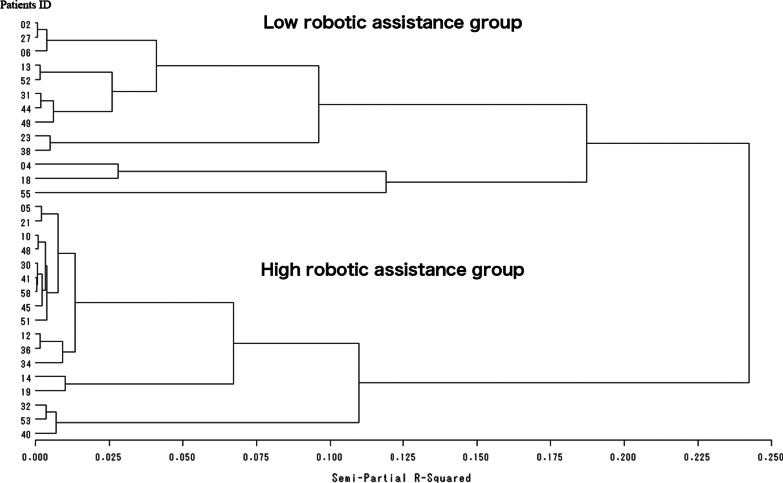
Dendrogram using Ward linkage. A dendrogram of the two identified distinct clusters based on the hierarchical cluster analysis using the Ward’s method

Results of the cluster analysis and characteristics of the two groups are displayed in Fig. 3 and Table 1, respectively. Two clusters were determined to be appropriate in view of the number of subjects overall, the number of subjects in the cluster, and the distance of cluster joining. Cluster analysis was used to assign the 30 participants in the robotic group into the following two groups: the high robotic assistance group (including 17 participants who were asked to conduct voluntary movement training with high assistance from a robot) and the low robotic assistance group (including 13 participants who were asked to conduct voluntary movement training with low assistance from a robot) (Table 2). The following significant differences were observed between the high and low robotic assistance groups in terms of the number of times that each level of robotic assistance was used: guided (high versus low robotic assistance group: 849.4 ± 247.0 versus 508.3 ± 118.1, p < 0.001); initiated (388 ± 336.5 versus 457.4 ± 264.5, p = 0.267; step-initiated (13.7 ± 22.3 versus 223.9 ± 165.0, p < 0.001); follow-assist (1.0 ± 3.1 versus 41.2 ± 57.9), p = 0.008; and free (2.5 ± 2.5 versus 7.1 ± 15.4, p = 0.238) modes. Therefore, the high and low robotic assistance groups were clearly differentiated in terms of the number of times using each level of robotic assistance was used.

**Table 1:** Characteristics of patients in the high and low robotic assistance groups

	High robotic assistance group (N = 17)	Low robotic assistance group (N = 13)	p value
Sex (male/female)	10/7	11/2	0.127
Age (years)	67.7 ± 9.6	62.0 ± 12.2	0.165
Weight (kg)	55.18 ± 7.38	61.32 ± 9.12	0.052
Height (cm)	160.65 ± 7.52	165.74 ± 6.35	0.06
Dominant hand (right/left)	17/0	13/0	1.00
Affected side (right/left)	5/12	5/8	0.602
Disability of the dominant hand (no/yes)	12/5	8/5	0.602
Days from stroke onset (days)	47.9 ± 7.1	47.6 ± 7.2	0.920
Classification of stroke causes (cardiogenic cerebral embolism/atherothrombotic stroke/lacunar infarction/others)	0/4/6/7	2/1/0/10	0.019
Categories of OCSP (LACI/TACI/PACI/POCI)	5/0/12/0	2/3/8/0	0.099
Score of Brunnstrom recovery stage at baseline (stage III/IV)	11/6	8/5	0.858
Concomitant medications and rehabilitation approaches (no/yes)	0/17	0/13	1.00

Data are described as mean ± standard deviation

*OCSP* Oxford Community Stroke Project, *LACI* lacunar infarct, *TACI* total anterior circulation infarct, *PACI* partial anterior circulation infarct, *POCI* posterior circulation infarct

**Table 2:** Comparison of assistance levels for voluntary movement training at each mode in the two groups

	High robotic assistance group	Low robotic assistance group
Guided mode	68% of the entire intervention time	41% of the entire intervention time
Initiated mode	31% of the entire intervention time	37% of the entire intervention time
Step-Initiated mode	1% of the entire intervention time	18% of the entire intervention time
Follow-assist mode	0%	3% of the entire intervention time
Free mode	0%	1% of the entire intervention time

See Fig. 2 for an interpretation of the levels of assistance for each mode of the robotic system

## Results

### Efficacy outcomes

Firstly, the normality of the amount of change in each outcome was ensured as follows (FMA, p = 0.709; FMA-proximal, p = 0.084, WMFT-PT, p = 0.397; WMFT-FAS, p = 0.784). The amount of change between pre- and post-intervention in each outcome was compared between the low and high robotic assistance groups, considering each severity class as well (Table 3).

**Table 3:** Severity of UE in each impairment class in the high and low robotic assistance groups

	Severity of UE hemiplegia at baseline	Group	Pre-intervention	Post-intervention	Amount of change	p-value of two-way ANOVA (severity × group) in between-group comparisons (effect size)
FMA	FMA < 30	High RA group (N = 12)	14.8 ± 7.0	27.4 ± 13.2	12.7 ± 9.8	0.103 (η^2^ = 0.09)
		Low RA group (N = 5)	20.0 ± 5.6	28.0 ± 7.8	8.0 ± 4.0	
	FMA > 30	High RA group (N = 5)	44.8 ± 5.2	48.6 ± 8.1	3.8 ± 5.8	
		Low RA group (N = 8)	46.6 ± 6.8	55.0 ± 2.9	9.3 ± 6.2	
FMA-proximal (shoulder/elbow/forearm)	FMA < 30	High RA group (N = 12)	10.8 ± 5.5	18.6 ± 7.5	7.8 ± 5.4	0.038 (η^2^ = 0.13)
		Low RA group (N = 5)	15.2 ± 3.6	19.0 ± 4.7	3.8 ± 3.3	
	FMA ≥ 30	High RA group (N = 5)	27.4 ± 3.4	27.6 ± 3.2	0.2 ± 1.5	
		Low RA group (N = 8)	27.4 ± 4.7	31.1 ± 2.9	3.8 ± 4.3	
WMFT-PT	FMA < 30	High RA group (N = 12)	1466.2 ± 266.4	1137.3 ± 357.1	− 328.9 ± 298.4	0.021 (η^2^ = 0.17)
		Low RA group (N = 5)	1524.0 ± 226.6	1280.8 ± 241.3	− 242.0 ± 51.0	
	FMA ≥ 30	High RA group (N = 5)	434.8 ± 289.1	5511.8 ± 378.3	77.0 ± 233.0	
		Low RA group (N = 8)	477.6 ± 301.7	-341.9 ± 292.7	− 341.9 ± 292.7	
WMFT-FAS	FMA < 30	High RA group (N = 12)	17.1 ± 9.1	26.3 ± 12.7	9.3 ± 13.3	0.045 (η^2^ = 0.14)
		Low RA group (N = 5)	18.2 ± 8.6	22.4 ± 8.7	4.2 ± 8.2	
	FMA ≥ 30	High RA group (N = 5)	44.0 ± 12.9	43.6 ± 15.1	− 0.4 ± 8.3	
		Low RA group (N = 8)	44.9 ± 4.9	56.9 ± 0.7	11.4 ± 6.0	

Data are presented as mean ± SD

*UE* upper extremity, *FMA* Fugl-Meyer Assessment, *RA* robotic assistance, *WMFT* Wolf Motor Function Test, *PT* performance time, *FAS* Functional Assessment Scale, *ANOVA* analysis of variance

The amount of change in FMA score between pre- and post-intervention in the group with a severe-to-moderate class was as follows: 12.7 ± 9.8 points in the high robotic assistance group and 8.0 ± 4.0 points in the low robotic assistance group. Contrarily, the moderate-to-mild class of both groups scored as follows: 3.8 ± 5.8 points in the high robotic assistance group and 9.3 ± 6.2 points in the low robotic assistance. In the two-way ANOVA, the group × severity interaction was not significantly different in each group (p = 0.103).

For the amount of change in FMA-proximal score between pre- and post-intervention, the patients with severe-to-moderate class receiving high robotic assistance showed an improvement of 7.8 ± 5.4 points and those receiving low robotic assistance had an improvement of 3.8 ± 3.3 points. On the other hand, subjects with moderate-to-mild class receiving high robotic assistance showed an improvement of 0.2 ± 1.5 points, whereas those receiving low robotic assistance had an improvement of 3.8 ± 4.3 points. The two-way ANOVA indicated significant difference in the group × severity interaction (p = 0.038).

For the amount of change in WMFT-PT score between pre- and post-intervention, patients with severe-to-moderate class receiving high robotic assistance showed an improvement of 328.9 ± 298.4 s after the intervention process, whereas those receiving low robotic assistance had an improvement of 242.0 ± 51.0 s. In contrast, patients with the moderate-to-mild class in both groups scored as follows: 77.0 ± 233.0 s in the high robotic assistance group; and 341.9 ± 292.7 s in the low robotic assistance group. A two-way ANOVA showed significant difference in the group × severity interaction (p = 0.021).

The amount of change in WMFT-FAS score between pre- and post-intervention in patients with severe-to-moderate class was as follows: 9.3 ± 13.3 points in the high robotic assistance group and − 4.2 ± 8.2 points in the low robotic assistance group. On the other hand, the change in WMFT-FAS score in patients with moderate-to-mild class was as follows: − 0.4 ± 8.3 points for the high robotic assistance group and 11.4 ± 6.0 points for the low robotic assistance group. A significant difference of group × severity interaction was shown in the two-way ANOVA (p = 0.045).

## Discussion

This research was performed to investigate how the level of robotic assistance and its adjustment could affect the functional improvement of the affected UE in two stroke survivor groups from the previous study [19]: one group with severe-to-moderate UE hemiplegia; and the other group with moderate-to-mild UE hemiplegia. This study analyzed the outcome of two groups (the intervention group receiving “considerable” robotic assistance and the control group receiving “minimal” robotic assistance). The findings in this study demonstrated that the intervention group comprising stroke survivors with severe-to-moderate stroke severity (FMA < 30) showed more significant improvement in UE hemiplegia and performance (measured using FMA-proximal, WMFT-PT, and WMFT-FAS) than the control group with the same severity of UE condition. Interestingly, the stroke survivors with moderate-to-mild stroke severity (FMA** ≥ **30) in the control group showed significantly improved UE hemiplegia and UE performance measured using the same outcome tools compared to the intervention group.

Regarding the group × severity interaction, there was no significant difference found in the FMA score. This could be because of the fact that the ReoGo robot used in this study is a type of robot that immobilizes the paralyzed hand but assists in exercises involving the shoulder, elbow, and forearm only [23]. More specifically, we believe that FMA, which does not assess the outcome of the paralyzed shoulder, elbow, and forearm, unlike the FMA-proximal, might have indicated more reliable and accurate results considering the way we utilized the ReoGo in this study.

This result suggests that, to maximize the improvements of the affected UE function in sub-acute stroke survivors, the robotic assistance may need to be increased in patients with severe-to-moderate paralysis and decreased in patients with moderate-to-mild paralysis, to improve the performance of voluntary movements as much as possible.

The American College of Cardiology/Stroke Society [6] suggests that robotic therapy can allow stroke survivors with moderate-to-severe upper limb paraplegia to participate in intensive practice. Furthermore, assistance from a robot is suggested to be implemented only as needed, and this approach is proved to be effective for facilitating UE recovery, especially in those with moderate-to-severe UE impairment. Considering recent trends of motor learning and neurorehabilitation [24, 25], minimal robotic assistance has been preferred to be implemented in stroke rehabilitation. Some studies [24, 26] highlighted that providing minimal robotic assistance can facilitate improvement of UE function by reducing spasticity of the affected UE in stroke survivors. Additionally, passive repetitive robot-assisted training at a high-frequency level and conventional therapy were found to produce similar effects [24, 26] in terms of the improvement of UE function [25]. Therefore, based on the findings of the present and previous studies, the implementation of robotic assistance with an as-needed approach should be encouraged when during robotic therapy, with consideration of the level of voluntary movement that a stroke survivor with UE paraplegia can perform. However, few studies have investigated the effect of different levels of robotic assistance on UE function recovery.

One of the examples is a study by Rowe et al. [16] that compared the effects of high and low assistance levels generated by an “exoskeleton robot” aimed at improving the movement of the hand and finger on the affected UE in stroke survivors during the recovery phase. In this study, although there was no significant difference in the primary outcome of hand and finger recovery (measured using the Box and Block Test), the secondary outcomes of lateral pinch strength and UE (shoulder/elbow/forearm) function (based on FMA) appeared to significantly improve with higher assistance level from a exoskeleton robot. This study also reported that the amount of improvement in the affected hand function was greater in subjects with a more severe condition. This result was similar to some of our study findings. However, our study used an “end-effector robot” designed to improve UE (shoulder/elbow/forearm) function significantly improved the whole impairment in stroke survivors by providing appropriate level of assistance in each condition. A recent study involving patients with stroke reported that end-effector-type robotics were more effective for affected UE performance than exoskeleton-type robotics [27]. However, Moggio et al. [28] reported that more significant functional improvements were observed when exoskeleton-type robotics were used than when end-effector-type robotics were used for the affected finger in patients with stroke. Therefore, the effects of end-effector or exoskeleton-type robotics on the affected UE or affected fingers after stroke may vary. Thus, it is possible that the results of this study could be interpreted as novel as compared with those of previous studies.

Additionally, with regard to the detailed examination of the relationship between subjects’ severity of UE condition and robotic assistance in our study, the following findings were more evident in our study than in a previous study: the amount of assistance by the robot should be increased for subjects with severe-to-moderate UE impairment, and the amount of assistance by the robot should be reduced for subjects with moderate-to-mild UE impairment. The results of our study highlights the importance of considering “the ability to provide only the minimum assistance necessary”, which is one of the three hypotheses (high mechanical compliance, the ability to assist patients in completing desired movements, and the ability to provide only the minimum assistance necessary) stated by Wolbrecht et al. [29] for stroke survivors to perform effective movement practice. Our study findings support this hypothesis; therefore, maximal improvement of the paralyzed UE could be increased when clinicians provide the right amount of robotic assistance level, considering the severity of the UE paralysis.

One of the mechanisms of the effect of active assistive exercise using a robot is Hebbian plasticity, which enhances the proprioceptive input associated with spontaneous movement of the affected UE. Takahashi et al. [30] reported that active assistive exercise using a robot significantly improved the function of the affected UE after stroke. In the same paper, they also reported that functional MRI evaluation of the grasping task practiced during active assistive exercise with a robot showed a significant increase in the activation of the sensorimotor cortex during the intervention period.

The results of our study would suggest that stroke survivors with severe-to-moderate UE impairment can have a reduced risk of error occurrence by increasing the amount of robotic assistance. Our study also suggested that stroke survivors with moderate-to-mild UE impairment can have a reduced risk of experiencing the Slacking Hypothesis by appropriately reducing the robotic assistance. Our study results would support the hypothesis that the assistance-as-needed active assistive exercise of the paralyzed UE generated by the robot facilitated the proprioceptive input, which caused Hebbian plasticity. However, it is still early to confirm whether the robotic assistance-as-needed approach for performing active assistive exercise of paralyzed UE is associated with Hebbian plasticity. Further study needs to include neurological evaluation of Hebbian plasticity, including functional MRI.

This study had several limitations. Data from a previous work [9] were utilized in the present study, but no explanation was provided for the exclusion of 655 patients; thus, there may have been a bias in the data analysis. Another limitation of the study was the small sample size. The small study population may have affected the subgroup analysis. In addition, there were some scattered P-values of < 0.05 in this study, but these were not adjusted for multiplicity. Therefore, this result might be incidental. Further, since this study was a retrospective sub-analysis of a previous study; thus, it remains unclear whether an appropriate level of robotic assistance promoted the functional improvement or whether the appropriate level of robotic assistance was determined after functional recovery. Therefore, no clear causal relationship was established between the amount of robotic assistance and amount of functional improvement, and our results should be interpreted while considering these factors. In addition, the amount of robot assistance according to the severity of the UE paralysis was examined using only the evaluated values based on classical clinical tests, but no examination using robot-specific objective parameters was conducted.

Therefore, future randomized controlled trials that use state-of-the-art robotic devices and that evaluates a larger sample size need to be conducted to investigate how the level and methodology of robotic assistance could impact the improvement of UE function.

## Conclusions

An optimal amount of robotic assistance was found to be a key to maximize improvement in post-stroke UE paralysis. Furthermore, the severity of UE paralysis is an important consideration when deciding the amount of assistance in robotic therapy. More specifically, to obtain maximum improvement with robotic rehabilitation in patients with UE paralysis after a stroke, it is important to set a lower robotic assistance level for mildly paralyzed patients and a higher robotic assistance level for severely paralyzed patients to encourage appropriate voluntary movements. In the future, it would be worthwhile to compare the effects of robot rehabilitation between low and high assistance groups through prospective randomized controlled trials.

## Data Availability

The datasets are available from the corresponding author on reasonable request.

## Abbreviations

FAS: Functional assessment scale
FMA: Fugl-Meyer Assessment
LACI: Lacunar infarct
OCSP: Oxford Community Stroke Project
PACI: Partial anterior circulation infarct
POCI: Posterior circulation infarct
PT: Performance time
RA: Robotic assistance
RCT: Randomized controlled trial
TACI: Total anterior circulation infarct
UE: Upper extremity
WMFT: Wolf motor function test
